# Prevalence and Predictors of Cerebral Microangiopathy Determined by Pulsatility Index in an Asymptomatic Population From the ILERVAS Project

**DOI:** 10.3389/fneur.2021.785640

**Published:** 2021-12-14

**Authors:** Francisco Purroy, Enric Sánchez, Albert Lecube, Gloria Arqué, Mikel Vicente-Pascual, Gerard Mauri-Capdevila, Núria Torreguitart, Marta Hernández, Ferrán Barbé, Elvira Fernández, Reinald Pamplona, Cristina Farràs, Dídac Mauricio, Marcelino Bermúdez-López, the ILERVAS project, José Manuel Valdivielso

**Affiliations:** ^1^Stroke Unit, University Hospital Arnau de Vilanova, Lleida, Spain; ^2^Clinical Neurosciences Group, IRBLleida, University of Lleida, Lleida, Spain; ^3^Endocrinology and Nutrition Department, University Hospital Arnau de Vilanova, Lleida, Spain; ^4^Obesity, Diabetes and Metabolism (ODIM) Research Group, IRBLleida, Lleida, Spain; ^5^University of Lleida, Lleida, Spain; ^6^CIBER de Diabetes y Enfermedades Metabólicas Asociadas, CIBERDEM, Instituto de Salud Carlos III (ISCIII), Madrid, Spain; ^7^Vascular Surgery Service, University Hospital Arnau de Vilanova, Lleida, Spain; ^8^Pneumology Service, Translational Research in Respiratory Medicine Research Group, IRBLleida, University Hospital Arnau de Vilanova, University of Lleida, Lleida, Spain; ^9^CIBER de Enfermedades Respiratorias, CIBERES, Instituto de Salud Carlos III (ISCIII), Lleida, Spain; ^10^Vascular and Renal Translational Research Group, IRBLleida, ReinRen-ISCIII, University of Lleida, Lleida, Spain; ^11^Department of Experimental Medicine, Metabolic Pathophysiology Research Group, IRBLleida, University of Lleida, Lleida, Spain; ^12^DAP Lleida, Unitat de Suport a la Recerca - IDIAP Jordi Gol, Lleida, Spain; ^13^Department of Endocrinology and Nutrition, CIBER of Diabetes and Associated Metabolic Diseases, Hospital de la Sant Creu i Sant Pau, Barcelona, Spain

**Keywords:** atheromatosis, cerebral microangiopathy, extracranial atheromatosis, pulsatility index (PI), transcranial Doppler (TCD), ultrasonography

## Abstract

**Background:** Little is known about the prevalence of cerebral microangiopathy (CM), which is related to cognitive impairment, in an asymptomatic population. Pulsatility index (PI) is an easily measurable parameter of cerebral vascular resistance in transcranial duplex of the middle cerebral artery (MCA) study. We aimed to determine the prevalence of CM measured by PI of MCA in low to moderate vascular risk subjects.

**Methods:** We included 3,721 subjects between 45 and 70 years without previous history of vascular disease or diabetes mellitus and with at least one other vascular risk factor from the cross-sectional study ILERVAS (Lleida, Spain). Patients underwent transcranial duplex to determine MCA-PI. Possible CM was defined by MCA-PI >1.1. Carotid and femoral arteries ultrasound registration was done to determine the presence, the number, and the area of atheromatous plaques. Body mass index (BMI), pulse pressure (PP) and laboratory data were also recorded.

**Results:** 439 (11.8%) subjects were excluded due to the low quality of transcranial duplex images. Median age was 57 [IQR 52, 62] years. Possible CM was found in 424 (12.9%) subjects. CM patients had higher prevalence of plaques than non-CM (77.4 vs. 66.4%, *p* < 0.001). PI showed a positive linear correlation with the number of territories with plaques (*r* = 0.130, *p* < 0.001), and the total plaque area (*r* = 0.082, *p* < 0.001). The predictors of possible CM were the age, male gender, and PP.

**Conclusions:** In low-to-moderate vascular risk asymptomatic population, the proportion of abnormal brain microvascular bed determined by MCA-PI is not negligible. The planned 10-year follow-up will describe the clinical relevance of these findings.

## Introduction

Pulsatility index (PI) is an easily measurable parameter of cerebral vascular resistance in the transcranial duplex (TCD) of the middle cerebral artery (MCA) study. It is a surrogated marker of cerebral microangiopathy (CM) that reflects the resistance of the microvascular distal bed of the measured vessel (1–9). MCA-PI is significantly correlated with white matter lesions severity in magnetic resonance imaging (MRI) (2, 4–9). In type 2 diabetes mellitus patients PI reflects extracranial (10) and intracranial (3, 11) microvascular complications. Changes in the PI of the MCA have been proposed as a measure of the pleiotropic action mechanism of selective phosphodiesterase inhibitors (12). Patients with CM have a neurovascular dysfunction (13) that contributes to a mismatch between neural activity and provided oxygen and glucose (14). The neurovascular unit disbalance is related with impairment of the cerebral function, which could be clinically expressed as depression, cognitive, and gait impairments (13–19). Neurovascular dysfunction is observed in the early disease stage of Alzheimer's disease (20). As a result, CM is the main pathogenic finding in up to 45% of cases of dementia (16), and prevalence of CM increases with age (13, 15–17). Hypertension is the main modifiable risk factor (13, 15–17). However, CM remained asymptomatic several years until onset clinical manifestations in many cases. Up to 20% of asymptomatic elderly people have shown CM on magnetic resonance imaging (MRI) studies (16). Early identification of patients with subclinical CM would provide an opportunity to begin intensive effective treatment to avoid emergence of symptoms.

Limited information exists on the presence of CM in low to moderate vascular risk subjects and its association with extracranial and intracranial angiopathy (21, 22). In this context, we have designed a study to analyze data from the ILERVAS project (ClinicalTrials.gov Identifier: NCT03228459) to describe the prevalence of abnormal PI, as a surrogated marker of CM, in an asymptomatic population (23, 24).

## Materials and Methods

### Ethics Statement

Informed consent was obtained from all participants. The protocol was approved by the Hospital Universitari Arnau de Vilanova's ethics committee (CEIC-1410).

### Study Design and Participants

The rationale and design of the ILERVAS study have been extensively described and baseline data was published (www.elbusdelasalut.cat) (23, 24). Briefly, for the current study, 3,721 people attended by a mobile unit were enrolled between September 2015 and December 2017 from Primary care centers across the entire province of Lleida, in West Catalonia (Spain). The inclusion criteria were: between 45 and 70 years old, without previous history of vascular disease or diabetes mellitus and with at least one other vascular risk factor (obesity, hypertension, dyslipidemia, smoking, or a first degree relative with premature cardiovascular disease). Subjects with diagnosed diabetes, chronic kidney disease, history of vascular events (ischemic disease, stroke, or peripheral arteriopathy), active neoplasia, a life expectancy of <18 months, and pregnancy were excluded.

Sociodemographic variables and anthropometrical data were registered. Weight and height were measured to determine body mass index (BMI, kg/m2). Neck and waist circumferences were measured in centimeters. Blood pressure was measured 3 times with an Omron-M6 apparatus at 2-min intervals, and the mean of the last two measurements calculated. A pulse pressure, the difference between systolic and diastolic pressures, was calculated. The pack-year smoking history and smoking status (non-smoker/current/former smoker) were also recorded. Smokers who stopped smoking ≥1 year prior to recruitment were considered former smokers. A dry analytical capillary blood sample to determine levels of glycosylated hemoglobin, total cholesterol, and serum creatinine was used. A urine sample was collected to determine creatinine levels and further estimated glomerular filtration rate (GFR) by the chronic kidney disease epidemiology collaboration (CKD-EPI) equation (25).

### Cerebral Microangiopathy

All patients underwent transcranial duplex (TCD) examinations to assess intracranial circulation. The ultrasound protocol has been previously published (23). Two experienced examinators performed the examinations according to consensus recommendations for an optimal exploration (26), using General Electric Vivid I/Pro equipment (Horten, Norway). Experienced neurologists (FP MV GMC) directly reviewed the first 50 explorations. Afterwards, only explorations with doubts were reviewed. The transcranial arteries were explored *via* transtemporal and transforaminal windows. The gosling PI of each MCA was calculated automatically by the equation [(systolic velocity-diastolic velocity)/mean velocity] (27). To ensure accurate PI values, we only considered measurements if the Doppler spectrum wave-form envelope showed good quality, in at least, four consecutive waveforms. Mean PI from both M1 segments of MCA was calculated. Possible CM was defined by MCA-PI >1.1 (27).

### Extracranial Arterial Ultrasound Study

All subjects underwent a bilateral carotid and femoral ultrasound exploration to assess the presence of atheromatous disease. Carotid exploration included common artery, bifurcation, internal, and external areas, whereas femoral exploration included common and superficial areas.

Plaques were defined as focal intrusions into the lumen ≥1.5 mm thick according to Mannheim intima-media thickness consensus (28). The area of each plaque was quantified with a caliper. The total plaque area was determined and expressed in cm^2^.

### Statistical Analysis

Comparisons between groups were performed using the Mann–Whitney *U* Test for numerical variables, and the Pearson's chi-squared for categorical variables. The correlation between quantitative variables was analyzed using Spearman's test. Quantitative data are showed in median (interquartile range [IQR]). Qualitative data are given in absolute and relative frequencies). The accuracy of pulse pressure as a measurement of interest to discriminate CM-patients from non-CM subjects was evaluated using a Receiver Operating Characteristic (ROC) curve analysis and a complete sensitivity/specificity report performed. The total area under the ROC curve value was interpreted following these scores: 0.9–1.0 = excellent; 0.8–0.9 = good; 0.7–0.8 = fair; 0.6–0.7 = poor; 0.5–0.6 = fail. A stepwise multivariate regression analysis was used to explore the variables independently related to PI. Variables with *p* < 0.10 in univariate testing were included. Statistical analyses were done using SPSS Statistics for Windows (version 20.0; IBM, Armonk, NY). Significance was considered at *p* < 0.05.

### Data Availability Statement

Request to access the data reported in this article will be considered by the corresponding author.

## Results

A total of 3,721 subjects were initially evaluated. A number of subjects, 439 (11.8%), were excluded due to low quality of TCD images. Therefore, 3,282 subjects were included in the study. Median age was 57 [IQR 52, 62] years and body mass index (BMI) was 28.6 [IQR 25.8, 31.8] kg/m^2^. The complete anthropometrical data, vascular risk factor diagnosis, smoking habits, laboratory data, and prevalence of atheroma plaque according to the presence of possible CM are shown in Table 1. There was a significant difference in all data between normal and abnormal MCA-PI, except for BMI and dyslipidemia diagnosis.

**Table 1:** Anthropometrical data, cardiovascular risk factor criteria, smoking habit, laboratory results, and prevalence of atheroma plaque from the ILERVAS population according to cerebral microangiopathy.

	**All population**	**Possible CM patients**	**Non-CM patients**	***p*-value**
**Anthropometrical data**				
n	3,282	424	2,858	
Sex (male), *n* (%)	1,719 (52.4)	242 (57.1)	1,477 (51.7)	0.042
Age (years)	57 [52;62]	61 [56;65]	56 [52;61]	<0.001
BMI (Kg/m^2^)	28.6 [25.8;31.8]	28.9 [26.3;32.2]	28.5 [25.6;31.7]	0.060
Waist circumference (cm)	100 [93;108]	102 [94;110]	100 [93;108]	0.001
Neck circumference (cm)	38 [35;41]	39 [36;42]	38 [35;41]	0.001
Systolic BP (mm Hg)	131 [120;142]	139 [127;151]	130 [119;141]	<0.001
Diastolic BP (mm Hg)	82 [76;88]	79 [73;85]	83 [76;89]	<0.001
Pulse pressure (mm Hg)	58 [41;57]	59 [50;69]	47 [40;55]	<0.001
**Vascular risk factor**				
Hypertension, *n* (%)	1,315 (40.1)	235 (55.4)	1,080 (37.8)	<0.001
Dyslipidemia, *n* (%)	1,686 (51.4)	217 (51.2)	1,469 (51.4)	0.932
**Smoking habit**				<0.001
Non-smoker, *n* (%)	1,204 (36.7)	186 (43.9)	1,018 (35.6)	
Current smoker, *n* (%)	985 (30.0)	89 (21.0)	896 (31.4)	
Former smoker, *n* (%)	1,093 (33.3)	149 (35.1)	944 (33.0)	
Tobacco packages per year	20.8 [9.8;33.9]	23.8 [10.6;38.4]	20.5 [9.6;33.3]	0.051
**Laboratory data**				
HbA1c (%)	5.5 [5.3;5.7]	5.5 [5.3;5.8]	5.5 [5.3;5.7]	0.001
Total cholesterol (mg/dL)	202 [180;227]	196 [177;222]	203 [181;229]	0.001
GFR (mL/min per 1.73 m^2^)	96.9 [87.8;103.3]	94.5 [83.9;100.8]	97.4 [88.3;103.6]	<0.001
Urine ACR (mg/g)	29 [29;29]	29 [29;29]	29 [29;29]	0.554
**Atheroma plaque**				
Anywhere, *n* (%)	2,226 (67.8)	328 (77.4)	1,898 (66.4)	<0.001
Only in carotid arteries, *n* (%)	446 (14.2)	67 (15.8)	399 (14.0)	0.312
Only in femoral arteries, *n* (%)	777 (23.7)	88 (20.8)	689 (24.1)	0.128
Affected territories	1 [0;3]	2 [1;4]	1 [0;3]	<0.001
Total plaque area (cm^2^)	0.5 [0.2;1.1]	0.6 [0.2;1.3]	0.5 [0.0;1.0]	0.014

*Quantitative data are expressed as median [interquartile range] and qualitative variables are n (percentage). CM, cerebral microangiopathy; BMI, body mass index; BP, blood pressure; HbA1c, glycosylated hemoglobin; GFR, glomerular filtration rate; ACR, albumin to creatinine ratio*.

**Figure 1:**
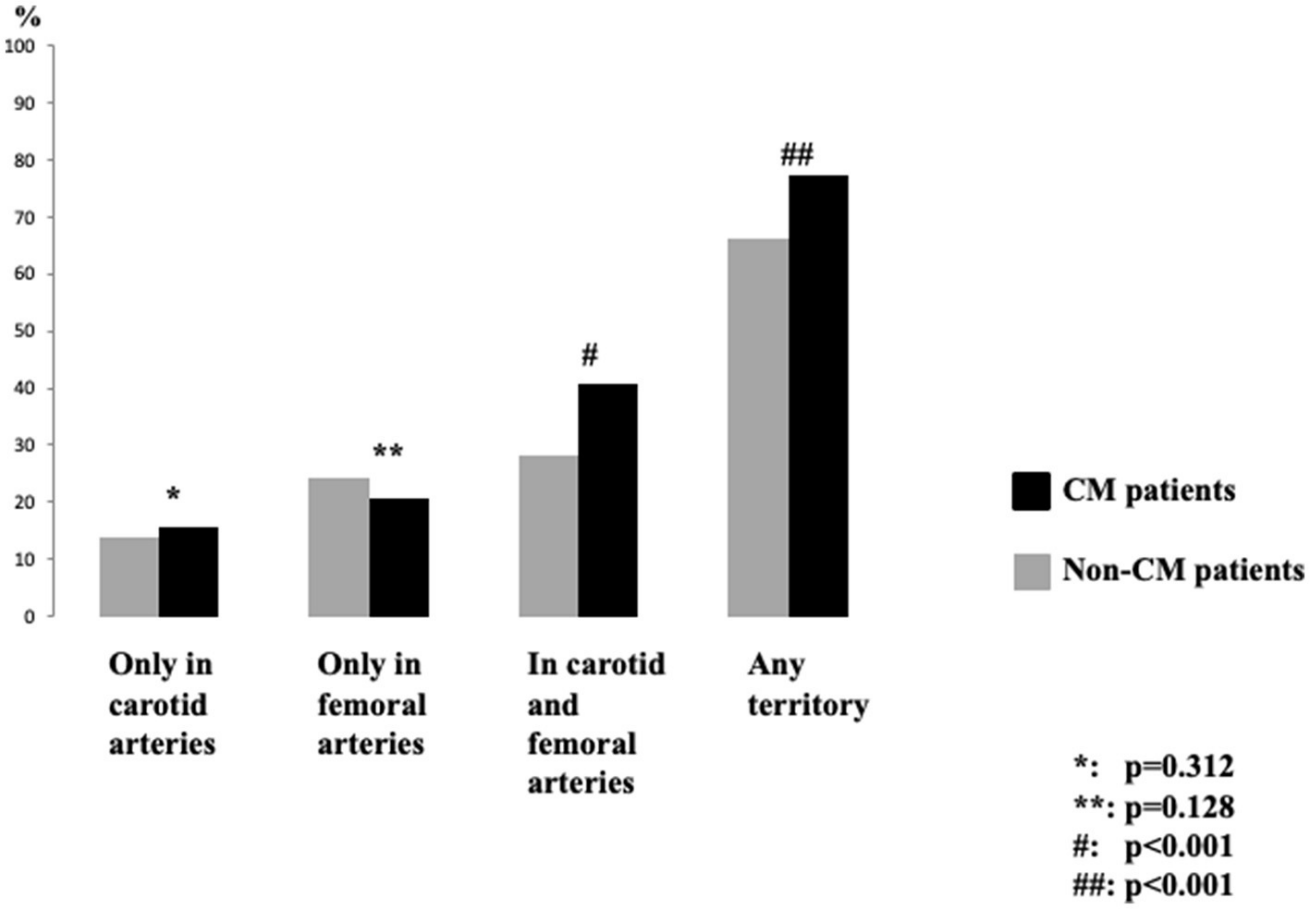
Prevalence of atheromatosis between cerebral microangiopathy patients according to the location of atheroma plaque. CM, cerebral microangiopathy.

Median PI value in all population was 0.84 [IQR 0.76, 0.93]. Possible CM was found in 424 (12.9%) subjects. Possible CM prevalence was increased according to age (STATS, *p* < 0.001). CM patients had higher prevalence of plaque number on affected territories and its total plaque area compared to non-CM patients (Table 1). Equal prevalence of atheromatosis in both groups was detected when carotid and femoral were analyzed separately (Figure 1). PI value had a positive linear correlation with several affected territories and its total plaque area in non-CM subjects. In addition, PI had a positive linear correlation with the number of territories with plaques (*r* = 0.130, *p* < 0.001), the total plaque area (*r* = 0.082, *p* < 0.001), the glycosylated hemoglobin levels (*r* = 0.065, *p* < 0.001), and a negative correlation with the glomerular filtration rate (*r* = −0.120, *p* < 0.001).

**Figure 2:**
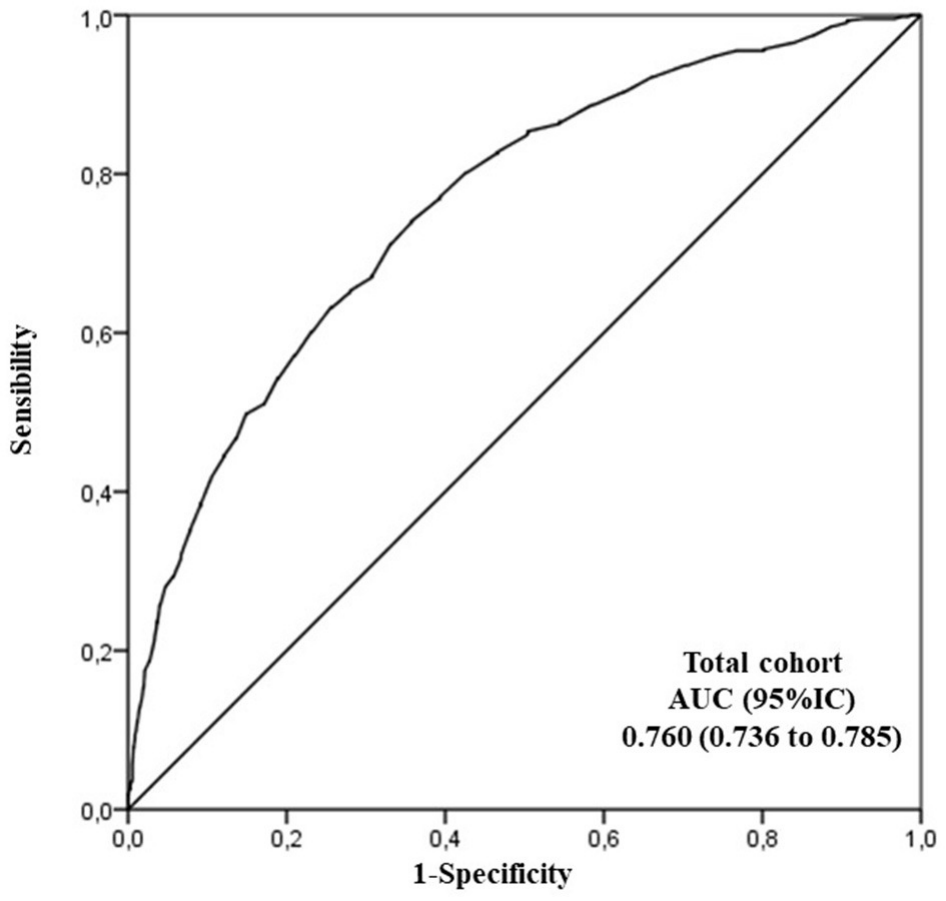
Receiver Operating Characteristics (ROC) curve analysis to evaluate the accuracy of pulse pressure as a measurement of interest to discriminate cerebral microangiopathy patients from non-cerebral microangiopathy together with a sensitivity/specificity report. AUC, area under the ROC curve.

Based on the ROC analysis, the pulse pressure was a good test to identify CM-patients among all subjects. The best cut-off point for pulse pressure (combining sensitivity and specificity) was 50 mmHg. At this value, the area under the curve was 0.760 (0.736–0.785), sensitivity was 74%, and specificity was 64% (Figure 2). Concretely, the percentage of CM-patients increased from 39.3% with a skin pulse pressure <50 mmHg to 76.9% of CM-patients with a skin pulse pressure ≥50 mmHg (STATS, *p* < 0.001). This data indicated a 5-fold increased risk of the presence of an abnormal PI [mean difference 5.1 (95% CI 4.1–6.5)] in comparison with subjects with normal PI values. Finally, a stepwise multivariate regression analysis showed that age, male gender, and pulse pressure were predictors of possible CM (STATS) (Table 2).

**Table 2:** Multinomial Logistic Regression model for presence of possible cerebral microangiopathy for development cohort.

		**OR (95% IC)**	***p***
Gender	Female	Ref.	
	Male	2.21 (1.49–3.25)	**<0.001**
Age (years)		1.04 (1.01–1.08)	**0.008**
BMI (kg/m^2^)		0.99 (0.96–1.02)	0.462
PP (mmHg)		1.07 (1.06–1.08)	**<0.001**
Hypertension Diagnosis	No	Ref.	
	Yes	0.89 (0.64–1.23)	0.471
Dyslipidemia Diagnosis	No	Ref.	
	Yes	0.89 (0.69–1.29)	0.719
Never smoker	Never	Ref.	
	Former	-	
	Current	-	
Packages per year		1.00 (0.99–1.01)	0.295
HbA1c (%)		1.33 (0.99–1.78)	0.056
Total cholesterol (mg/dL)		0.99 (0.99–1.00)	**0.021**
GFR (mL/min per 1.73 m^2^)		1.00 (0.99–1.01)	0.702
Atheroma plaque	No	Ref.	
	Yes	0.84 (0.55–1.28)	0.414

*BMI, body mass index; PP, pulse pressure; HbA1c, glycosylated hemoglobin; GFR, glomerular filtration rate. Bold indicates significant p-values*.

## Discussion

In the current study, we have observed that one of ten patients from a cohort of middle-aged population with low-to-moderate vascular risk showed possible CM detected by abnormal MCA-PI. Abnormal MCA-PI was related to sex male, age, pulse pressure, and the extension of the subclinical atheromatous. Traditionally, modifiable vascular risk factors (VRF) and age are both associated with atheromatous vascular disease which is the main leading cause of mortality worldwide (29). Moreover, according to epidemiological studies VRF contribute to cognitive decline (30). Treatment of VRF is associated with a reduced risk of Alzheimer's disease conversion in mild cognitive patients (31). Initially, the ILERVAS study was designed as a randomized, interventional, longitudinal clinical trial to assess the prevalence, vascular distribution, severity and progression of subclinical atheromatosis, and its impact on the incidence of vascular events on a 10-year follow-up (23). However, among low-to-moderate vascular risk patients it is also interesting to detect subclinical CM which could be related to cognitive impairment (13–15, 17, 32, 33). Previous studies have shown an elevation in PIs with small vessel disease (2–5) and cognitive impairment, or its progression (1, 32–34). In the Barcelona-Asymptomatic Intracranial Atherosclerosis (AsIA) study, which included 50–65 year-old subjects free from dementia and without history of vascular disease, MCA-PI was significantly associated with white matter disintegration in different tracts (fornix, corticospinal and anterior thalamic) measured by diffusion tensor images acquired on a 3T-MRI and with poor performance in attention, psychomotor speed, and visuospatial skills (21).

Our results are related to the previous evidence. We observed a relationship between age and abnormal MCA-PI. It is known that CM is directly related to age, and therefore, it is expected to observe a higher vascular resistance measured by MCA-PI in aged patients (13, 16, 17). Pulse pressure, which is associated with vascular dysfunction, has also been related to CM (35, 36). Interestingly, subjects with extended subclinical atheromatous disease with plaques in carotid and femoral territories had higher proportion of abnormal MCA-PI. So, in middle-aged subjects with low-to-moderate vascular risk macro and microvascular impairment could progress in parallel.

The main limitation of our study is that we have assumed that abnormal MCA-PI was a surrogated marker of CM, and we have accepted a cut-off previously established in a different population. We have realized that MRI is the preferred brain diagnostic imaging modality to detect CM (13, 15–17). However, TCD is widely used and less expensive than MRI. Finally, MCA-PI can be affected by cerebral hemodynamics like hypertensive or hypotensive status, anemia, or heart failure (2) that were not systematically recorded.

In conclusion, our data suggest that the proportion of subclinical potential CM is not insignificant. The clinical relevance of abnormal baseline MCA-PI should be determined after the end of the 10-year follow-up of the project. Our hypothesis is that abnormal baseline MCA-PI could increase the risk of brain microangiopathy (37), lacunar stroke, cognitive impairment, and motor impairment.

## Data Availability Statement

The raw data supporting the conclusions of this article will be made available by the authors, without undue reservation.

## Ethics Statement

The protocol was approved by the Arnau de Vilanova University Hospital Ethics Committee (CEIC-1410). The patients/participants provided their written informed consent to participate in this study.

## ILERVAS Project

José Manuel Valdivielso, Vascular and Renal Translational Research Group, IRBLleida, ReinRen-ISCIII, University of Lleida, Lleida, Spain; Serafí Cambray, Vascular and Renal Translational Research Group, IRBLleida, ReinRen-ISCIII, University of Lleida, Lleida, Spain; Eva Castro, Vascular and Renal Translational Research Group, IRBLleida, ReinRen-ISCIII, University of Lleida, Lleida, Spain; Montserrat Martínez-Alonso, Applied Epidemiology Research Group, Lleida, Spain; Manuel Portero-otín, Department of Experimental Medicine, Metabolic Pathophysiology Research Group, Systems Biology and Statistical Methods for Biomedical Research Group, IRBLleida, University of Lleida, Lleida, Spain; Mariona Jové, Department of Experimental Medicine, Metabolic Pathophysiology Research Group, Systems Biology and Statistical Methods for Biomedical Research Group, IRBLleida, University of Lleida, Lleida, Spain; Ferran Rius, Department of Endocrinology and Nutrition, Research Group on Immunology and Metabolism, IRBLleida, University Hospital Arnau de Vilanova, University of Lleida, Lleida, Spain; Jessica González, Pneumology Service, Translational Research in Respiratory Medicine Research Group, IRBLleida, University Hospital Arnau de Vilanova, University of Lleida, Lleida, Spain; Silvia Barril, Pneumology Service, Translational Research in Respiratory Medicine Research Group, IRBLleida, University Hospital Arnau de Vilanova, University of Lleida, Lleida, Spain; Gerard Torres, CIBER de Enfermedades Respiratorias, CIBERES, Instituto de Salud Carlos III (ISCIII), Lleida, Spain; Pere Godoy, Departament de Salut, Secretaria de Salut Pública, Generalitat de Catalunya, Lleida, Spain; Eva Miquel, DAP Lleida, Unitat de Suport a la Recerca - IDIAP Jordi Gol, Lleida, Spain; Marta Ortega, DAP Lleida, Unitat de Suport a la Recerca - IDIAP Jordi Gol, Lleida, Spain; Esmeralda Castelblanco, Applied Epidemiology Research Group, Lleida, Spain; Josep Franch-Nadal, Applied Epidemiology Research Group, Lleida, Spain.

## Author Contributions

FP, AL, FB, EF, RP, CF, DM, and MB-L conceived the study and procured funding. FP designed the experiments. AL, GA, MV-P, GM-C, NT, MH, FB, and CF were responsible for the recruitment of patients and clinical data acquisition. GA, ES, AL, and FP conducted the sample processing and data analysis. ES, AL, and FP wrote the paper. All authors commented on and approved submission of this manuscript.

## Funding

This work was supported by grants from the Diputacio de Lleida, Instituto de Salud Carlos III (RETIC RD16/0009/0011) and Ministerio de Ciencia, Inovación y Universidades (IJC2018-037792-I). FP was supported by the Catalan Autonomous Government's *Agència de Gestió d'Ajuts Universitaris i de Recerca* (2017 *suport a les activitats dels grups de recerca* 1628). RP was supported by the Spanish Ministry of Science, Innovation, and Universities (grant RTI2018-099200-B-I00), and the Generalitat of Catalonia: Agency for Management of University and Research Grants (2017SGR696). This study was co-financed by FEDER funds from the European Union (A way to build Europe). IRBLleida is a CERCA Programme/Generalitat of Catalonia.

## Conflict of Interest

The authors declare that the research was conducted in the absence of any commercial or financial relationships that could be construed as a potential conflict of interest.

## Publisher's Note

All claims expressed in this article are solely those of the authors and do not necessarily represent those of their affiliated organizations, or those of the publisher, the editors and the reviewers. Any product that may be evaluated in this article, or claim that may be made by its manufacturer, is not guaranteed or endorsed by the publisher.
